# Ethics education among obstetrics and gynecologists in Saudi Arabia: a cross-sectional study

**DOI:** 10.1186/s12909-023-04824-x

**Published:** 2023-11-16

**Authors:** Noha Abed Althagafi, Ghiath Alahmad

**Affiliations:** 1 King Fahad National Guard Hospital, Riyadh, Saudi Arabia; 2 King Saud Bin Abdulaziz University for Health Sciences, Riyadh, Saudi Arabia; 3 King Abdullah International Medical Research Center, King Saud Bin Abdulaziz University for Health Sciences, Riyadh, Saudi Arabia

**Keywords:** Obstetrics and gynecology, Ethics education, Ethics training, Saudi Arabia

## Abstract

There are a lot of efforts to promote ethics education and training at an undergraduate and postgraduate level around the world, including in Saudi Arabia. However, there is still a lack of structured ethics education curricula in obstetrics and gynecology residency programs in Saudi Arabia. In the current scenario, where new ethical dilemmas are emerging, an ethics education that only focuses on teaching the ethical principles does not enhance the competence to deal with the ethical challenges in daily practice. This study evaluates the lack of ethics education and training in obstetrics and gynecology residency programs in Saudi Arabia. A cross-sectional survey of working obstetricians and gynecologists in Saudi Arabia was conducted among all working levels of doctors (the residents, registrars, consultants, and program directors) from various hospitals of Saudi Arabia. 391 practitioners responded to the survey, representing a response rate of 39.1%, including 257 (66.4%) females and 130 (33.6%) males. 74 (23.0%) received formal education (medical school = 35%; residency programs = 11.8%; sub-specialty programs = 4.3%; postgraduate programs = 7.4%), 85 (26.4%) received informal education (online training = 12.8%; conferences = 18.9%; courses and workshops = 13.3%; self-learning = 31.7% and daily practices = 20.7%), and 78 (19.95%) received no ethics education. Almost all the respondents had a positive attitude towards ethical principles, but a least percent were competent to deal with the ethical challenges. The bottom line of this survey is the imperativeness of reinforcement of formal ethics education in obstetrics and gynecology postgraduate programs in Saudi Arabia.

**Supplementary Information:**

The online version contains supplementary material available at 10.1186/s12909-023-04824-x.

## Introduction

Ethical principles and ethical challenges are an inevitable aspect of medical practice. But the problem arises on how to efficiently implement bioethics education to medical students [1]. Autonomy, beneficence, non-maleficence, and justice are the basic ethical principles that were proposed by Beauchamp and Childress in 1979 and have been implemented over the past 50 years [2]. Medical and technological advancements have created a wide array of new ethical challenges. Obstetricians and gynecologists, like other specialties, face increasing ethical challenges for a variety of reasons. These challenges may be due to medically advanced technologies such as sex selection [3, 4] or due to customs rooted in social and cultural practices such as consanguineous marriages [5, 6] and adolescent marriages [7]. In fact, obstetrics and gynecology physicians face many of these ethical challenges in their daily clinical practice, including the general medical ethical challenges such as respecting patients' autonomy, confidentiality, justice and others; as well as specific challenges related to the nature of the specialty itself. It touches upon sensitive issues that include but are not limited to familial relationships, reproduction, paternity issues and the status of the fetus [8]. Examples of these ethical dilemmas include the following: abortion for medical or social reasons [9–11], other feto-maternal conflicts [12], patients' rights to choose the mode of delivery for non-obstetric indications [13], reproduction technologies and their relationship with religion, egg donation [14–16], sperm banking [17], surrogacy motherhood [18, 19], genetic studies for inherited genes [20], cloning [21, 22], end-of-life decisions and advanced directives for pregnant women [23, 24] and many other challenges [25, 26]. Obstetrics and gynecology (OB/GNY) physicians must have sufficient knowledge to know how to deal with such situations.

It has become imperative to include ethics education in the curriculum of OB/GYN, as medical knowledge alone cannot enhance the competence to face ethical issues in daily practice [27]. Academic institutions, hospitals and training centers are few sources of ethics education. An effective and successful ethics education can only be achieved through training and mentoring, which can take various forms and types, and extend over years of study and practice. Bioethics theoretical education aims to provide proper guidance to address the ethical dilemmas faced in daily practice. The ethics education can be improved by including hands-on training, case-based studies in the curriculum and informal discussion with the faculties [28], action-based pedagogy and conflict management strategies [29]. Workbook-based ethics learning that includes ethics case discussions, classroom quizzes, and reflective writing are considered the most effective strategy for learning biomedical ethics [30]. A practical ethics curriculum structured by experienced professionals would enhance the competence to face ethical challenges [31]. Therefore it has become important to introduce medical ethics programs in medical institutions and treat medical ethics as not just a theoretical subject but as an important discipline of medical care [32].

Ethics education has also rapidly expanded in residency programs in several specialties (e.g., internal medicine, pediatrics, family medicine, and psychiatry) [33].In addition, there are many ethical medical educational components in the different residency training programs worldwide, including programs for OB/GYN. The Royale College of Obstetrics and Gynecology (RCOG) and The American College of Obstetrics and Gynecology (ACOG), as well as organizations from other countries, such as Canada, Australia, Japan, Malaysia and other countries, organize these training programs [34, 35]. Online training programs, conferences, courses and workshops, and self-learning are different modes of ethics education.

Many efforts and developments in bioethics are underway in Saudi Arabia, similar to those in many other countries. There are increasing numbers of ethics committees in hospitals and research centers. There is a growing interest in teaching medical ethics and training programs in Saudi Arabia at many levels. At the undergraduate level, many medical faculties have begun to include ethics education in their curricula, focusing mainly on the principles of ethics, informed consent and research ethics. Meanwhile, at the postgraduate level, some courses are related to ethics in general, particularly research ethics. A Master in Bioethics program also started at the University of King Saud bin Abdulaziz for Health Sciences in 2008 [36, 37].

This research is one of the few researches evaluating the level and type of ethics education, attitude toward ethical challenges and ethical principles among the OB/GYN physicians in Saudi Arabia.

## Methods

### Sample size

A cross-sectional survey of currently working obstetricians and gynecologists in Saudi Arabia. Our study included all working levels of doctors (the residents, registrars, and consultants) from various hospitals throughout the Kingdom of Saudi Arabia. No limits to gender, nationality or religion were applied in selecting participants. Monkey surveys (Emails, social media, WhatsApp, Facebook) were used as questionnaire delivery methods and reminder emails were sent three times a week. The link to the questionnaire was sent to 1000 OB/GYNs, of which 391 responded.

### Study setting

This study was conducted in numerous hospitals throughout Saudi Arabia, including King Abdulaziz Medical City, Internal Force Hospital, King Faisal Specialist Hospital, King Fahad Medical City, and King Khalid University Hospital. After conducting a pilot study, minor changes were made based on valuable inputs provided by physicians to finalize the questionnaire. The final questionnaire contained three segments. The initial segment of the questionnaire was divided into two parts. The first part was designed to acquire the demographic data of the participants and the second part was to acquire the information about the bioethics education and training. The next segment of the questionnaire focused on the bioethics principles. The last segment had questions related to the attitude toward the ethical challenges faced by OB/GYNs in daily practice. To develop a valid tool for measuring the attitude towards the ethical challenges and ethical principles of obstetricians and gynecologists, questionnaires were tested for validity and reliability through pilot testing prior to commencement. Several specialists from different medical specialties reviewed the questions to ascertain the suitability, effectiveness, and content validity. Test–retest reliability was ensured in a pilot study of 5 subjects prior to data collection. Participants' responses to each statement included, "Strongly agree", "Agree", "Neutral", "Disagree" or "Strongly disagree". Likert five–point scale was used. An official ethical approval was obtained from the IRB at King Abdulaziz Medical City prior to the conduction of the study.

### Sampling technique

A random cluster sampling technique was incorporated for respondents in the study.

### Data management and analysis plan

#### Statistical analysis

Assuming a prevalence of 50%, a 95% confidence level, a two-sided interval, and a five percent precision level, the optimal sample size required at the time of analysis was 391 cohorts. The sample size was calculated by using N-Query Advisor Version 4.0. The optimal sample size for estimating proportions was determined by using the Cochran formula.

#### Descriptive analysis

All variables were summarized and reported across the study cohorts using descriptive statistics. Interval variables such as age were summarized and reported in terms of mean and standard deviation. Categorical variables such as gender were summarized and reported in terms of frequency distribution.

#### Comparative analysis

All categorical and interval variables were compared statistically between aware and unaware subjects using the Chi-Square test for independence and one sample T-test, respectively. All statistical tests were considered significant at α level less than 0.05. SAS 9.2 was used for all statistical analyses.

#### Ethical considerations

An official approval for conducting this study was sought from the IRB. Following this, participants were contacted and asked about willingness to participate in our study. Written informed consent was obtained from each participant after clarification of the study objectives and activities. Confidentiality and privacy were respected and identical information was collected. Participants were advised to contact the primary investigator by email or phone for any further clarifications or inquiries.

## Result

### Descriptions of the characteristics of the respondents

A total of 391 out of 1,000 OB/GYN practitioners responded to the survey questions by email; therefore, the response rate was 39.1%. Participants responded from all provinces of Saudi Arabia. Female respondents totaled 257 (66.4%), which was almost double the rate of male respondents. The married respondents totaled 291 (75.6%), whereas 94 (24.4%) were unmarried.

The study included participants of all ages, with approximate percentages of the participants between 30 and 50 years is more than 60%.

Saudi physicians accounted for 213 (55.9%) participants and 371 (94.8%) Muslims. Approximately 247(63.1%) of the respondents were working in a tertiary government teaching hospital, whereas government non-teaching and private hospitals accounted for 107 (27%) of the participants.

Fifty-five percent of the participants were OB/GYN Board certified under different types of boards. Most of the physicians were certified by the Saudi Arabian board (18.2%), followed by the Arab board and Egyptian board (10.5%) and (6.9%) respectively; however, physicians holding Western certificates from Canada, England, US, or Indian boards were minimal in numbers.

The participants had equal percentages in relation to their tier position. The consultants and registrars in the sample numbers were 119 (30.4%) and 126 (32.23%), respectively; the remaining were residents.

Around 192 (49%) physicians had more than 10 years of experience in the field of OB/GYN. Currently, 61 (15.6%) of the practitioners face 1–10 ethical issues monthly in their practice, while the majority 309 (79.03%) face less than one issue per month (Table 1).

**Table 1:** Demographic & bioethics education against gender, marital status, age, nationality, and religion

Characteristics		Formal bioethics education					Informal bioethics education					Total
			**Medical school**	**Residency programs**	**Sub-specialty Programs**	**Postgraduate programs**	**Conferences**	**Online training**	**Courses & workshops**	**Daily practice**	**Self-learning**	
Gender	**Male**	**N**	49	16	12	11	26	24	20	32	50	130
		**%**	37.7	12.3	9.2	8.5	20	18.5	15.4	24.6	38.5	33.25
	**Female**	**N**	88	30	5	17	48	26	30	49	74	257
		**%**	34.2	11.7	1.9	6.6	18.7	10.1	11.7	19.1	28.8	65.73
	**Pearson Chi-Square** Table		0.503	0.855	0.001*	0.508	0.755	0.021*	0.304	0.205	0.054	-
Marital status	**Married**	**N**	84	33	16	27	54	34	44	72	86	291
		**%**	28.9	11.3	5.5	9.3	18.6	11.7	15.1	24.7	29.6	74.42
	**Single**	**N**	28	8	0	0	1	10	6	5	18	60
		**%**	46.7	13.3	0	0	21.7	16.7	10	8.3	30	15.35
	**Others**	**N**	23	4	1	0	7	6	2	4	20	34
		**%**	67.6	11.8	2.9	0	20.6	17.6	5.9	11.8	58.8	8.70
	**Pearson Chi-Square**		0	0.909	0.153	0.009*	0.837	0.405	0.226	0.007*	0.002	-
Age	** < 30**	**N**	41	10	0	1	15	13	6	10	24	71
		**%**	57.7	14.1	0	1.4	21.1	18.3	8.5	14.1	33.8	18.16
	**30–39**	**N**	52	19	4	11	28	14	15	28	48	140
		**%**	37.1	13.6	2.9	7.9	20	10	10.7	20	34.3	35.81
	**40–49**	**N**	30	13	9	7	17	14	18	25	33	104
		**%**	28.8	12.5	8.7	6.7	16.3	13.5	17.3	24	31.7	26.60
	** = > 50**	**N**	14	4	4	9	14	9	13	17	19	72
		**%**	19.4	5.6	5.6	12.5	19.4	12.5	18.1	23.6	26.4	18.41
	**Pearson Chi-Square**		0*	0.321	0.032*	0.083	0.855	0.402	0.167	0.389	0.685	-
Nationality	**Saudi Arabian**	**N**	88	21	12	8	46	30	3	40	71	213
		**%**	41.3	9.9	5.6	3.8	21.6	14.1	0.8	18.8	33.3	54.48
	**Non-Saudi Arabian**	**N**	47	23	5	20	27	19	8	40	50	168
		**%**	28	13.7	3	11.9	16.1	11.3	6.7	23.8	29.8	42.97
	**Pearson Chi-Square**		0.007*	0.245	0.212	0.002*	0.174	0.422	0.095	0.231	0.457	-
Religion	**Muslim**	**N**	128	39	15	27	70	42	50	79	116	371
		**%**	34.5	10.5	4	7.3	18.9	11.3	13.5	21.3	31.3	94.88
	**Non-Muslim**	**N**	8	5	1	0	3	6	1	1	6	13
		**%**	61.5	38.5	7.7	0	23.1	46.2	7.7	7.7	46.2	3.32
	**Pearson Chi-Square**		0.045*	0.002*	0.518	0.313	0.704	0*	0.546	0.235	0.257	-
Position	**Resident**	**N**	61	18	0	4	24	12	11	19	41	101
		**%**	60.4	17.8	0	4	23.8	11.9	10.9	18.8	40.6	25.83
	**Registrar/Specialist**	**N**	46	17	2	14	24	16	21	34	36	126
		**%**	36.5	13.5	1.6	11.1	19	12.7	16.7	27	28.6	32.23
	**Consultant**	**N**	29	11	15	10	26	22	20	27	47	119
		**%**	24.4	9.2	12.6	8.4	21.8	18.5	16.8	22.7	39.5	30.43
	**Pearson Chi-Square**		0*	0.174	0*	0.144	0.682	0.298	0.384	0.346	0.101	-
Board certification	**Arab Board**	**N**	11	4	3	3	8	7	5	3	15	41
		**%**	26.8	9.8	7.3	7.3	19.5	17.1	12.2	7.3	36.6	10.49
	**Saudi Board**	**N**	26	7	6	3	17	14	7	18	26	71
		**%**	36.6	9.9	8.5	4.2	23.9	19.7	9.9	25.4	36.6	18.16
	**Egyptian (MA, PhD)**	**N**	12	3	2	5	7	9	7	12	10	27
		**%**	44.4	11.1	7.4	18.5	25.9	33.3	25.9	44.4	37	6.91
	**Jordanian Board**	**N**	3	2	0	0	2	1	1	1	3	11
		**%**	27.3	18.2	0	0	18.2	9.1	9.1	9.1	27.3	2.81
	**Canadian Board**	**N**	1	1	0	1	0	1	2	3	2	5
		**%**	20	20	0	20	0	20	40	60	40	1.28
	**MRCOG**	**N**	4	3	1	0	5	4	2	6	8	17
		**%**	23.5	17.6	5.9	0	29.4	23.5	11.8	35.3	47.1	4.35
	**ABOG**	**N**	4	2	1	0	3	3	0	2	3	6
		**%**	66.7	33.3	16.7	0	50	50	0	33.3	50	1.53
	**Pearson Chi-Square**		0.387	0.759	0.815	0.064	0.177	0.029*	0.084	0.01*	0.818	-
Workplace	**Tertiary teaching hospital**	**N**	36	11	7	4	27	10	17	29	38	107
		**%**	33.6	10.3	6.5	3.7	25.2	9.3	15.9	27.1	35.5	27.37
	**Governmental teaching hospital**	**N**	61	15	3	9	27	16	13	26	51	140
		**%**	43.6	10.7	2.1	6.4	19.3	11.4	9.3	18.6	36.4	35.81
	**Private hospital**	**N**	25	6	6	9	11	16	11	14	22	55
		**%**	45.5	10.9	10.9	16.4	20	29.1	20	25.5	40	14.07
	**Governmental non-teaching hospital**	**N**	14	14	1	6	9	8	11	12	13	52
		**%**	26.9	26.9	1.9	11.5	17.3	15.4	21.2	23.1	25	13.30
	**Pearson Chi-Square**		0.084	0.015*	0.04*	0.025*	0.600	0.004*	0.095	0.43	0.388	-
Experience	** < 5**	**N**	36	8	0	5	15	7	9	16	23	62
		**%**	58.1	12.9	0	8.1	24.2	11.3	14.5	25.8	37.1	15.86
	**5–10**	**N**	42	17	1	5	23	12	9	19	33	89
		**%**	47.2	19.1	1.1	5.6	25.8	13.5	10.1	21.3	37.1	22.76
	** > 10**	**N**	56	20	16	18	35	30	31	45	66	192
		**%**	29.2	10.4	8.3	9.4	18.2	15.6	16.1	23.4	34.4	49.10
	**Pearson Chi-Square**		0*	0.134	0.005*	0.564	0.288	0.676	0.404	0.815	0.873	-
Religion	** < 1 per month**	**N**	116	31	12	19	53	37	33	51	90	309
		**%**	37.50	10.00	3.90	6.10	17.20	12.00	10.70	16.50	29.10	79.03
	**1–10 per month**	**N**	11	11	3	6	16	8	13	24	25	61
		**%**	18.00	18.00	4.90	9.80	26.20	13.10	21.30	39.30	41.00	15.60
	** > 10 per month**	**N**	10	4	2	4	5	5	6	6	9	21
		**%**	47.60	19.00	9.50	19.00	23.80	23.80	28.60	28.60	42.90	5.37
	**Pearson Chi-Square**		0.007*	0.118	0.458	0.068	0.214	0.29	0.009*	0*	0.101	-

^*^Significant at α level less than 0.05

### Bioethics education and training

Approximately 85 (21.7%) of the participants received mixed ethics education (formal ethics education and informal bioethics education), whereas 74 (18.9%) received only formal ethics education and 85 (21.7%) received only informal ethics education. In addition, 78 (19.95%) did not have any type of bioethics education.

**Fig. 1:**
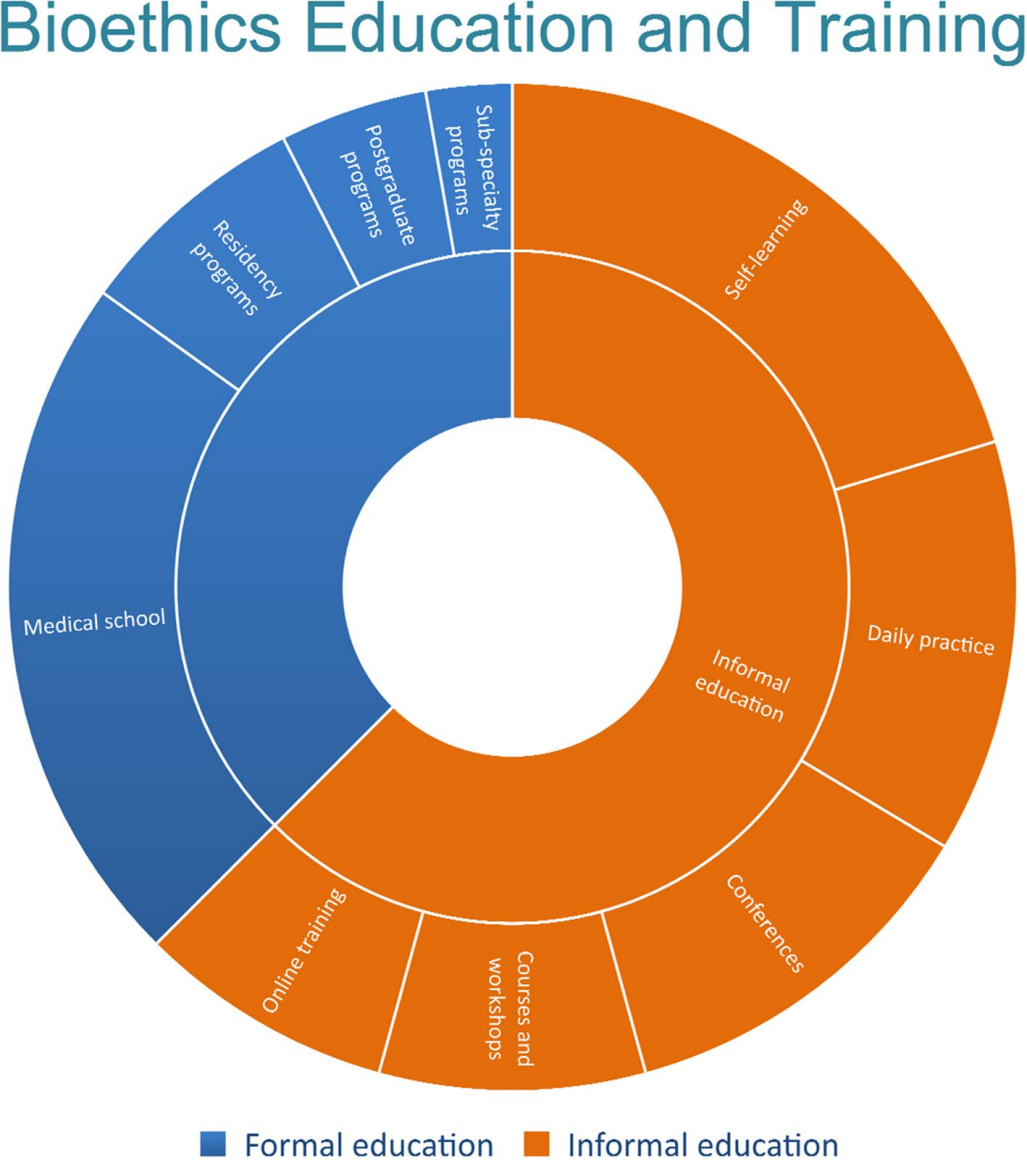
Modes of formal and informal bioethics education

Approximately 75% of the respondents received different types of formal and informal bioethics education. Of the respondents, 25% had no bioethics education; 137(35%) of physicians received a formal education during medical school; however, only 46 (11.8%) throughout residency programs. Self-learning was the method used for informal bioethics education in 124 (31.7%) cohorts (see Table 2 & Fig. 1).

**Table 2:** Bioethics education and training

Did you get an ethics education		*Yes*	%
Did you receive formal teaching in bio-medical ethics?	Only formal education without any other teaching	74	18.9
	Formal education with other informal teachings	85	21.7
	Formal educationin total	159	40.7
When did you get your formal education	In medical school	137	35.0
	During residency programs	46	11.8
	During sub-specialty programs	17	4.3
	In postgraduate programs	29	7.4
Did you receive informal teaching in bio-medical ethics?	Only informal education without any formal education	85	21.7
	Informal education with formal education	85	21.7
	Informal education in total	170	43.5
Where did you receive informal teaching in bio-medical ethics?	In conferences	74	18.9
	Online training	50	12.8
	In courses and workshops	52	13.3
	Daily practice (grand round, case presentation.)	81	20.7
	Self-learning	124	31.7

#### Type of bioethics education across demographic data

No differences related to gender or the type of ethics education received in medical school during residency programs, postgraduate programs, conferences, courses and workshops and daily practice were detected. However, male respondents, more so than female respondents, agreed to have received ethics education in sub-specialty programs. The same finding was true regarding self-learning and online training.

Regarding marital status and type of ethics education, no significant differences during residency programs, sub-specialty programs, in conferences, online training, in courses and workshops were found. Single respondents agreed to receive an ethics education in medical school compared to married people. Married respondents received a greater degree of informal bioethics education through daily practice and self-learning, while others received it through self-learning. There exists a significant difference between marital status and medical school *(P* = *0.00)*, postgraduate programs *(P* = *0.009)*, daily practice *(P* = *0.007)* and self-learning *(P* = *0.002)*.

Regarding age, no significant differences were found in ethics education, except in medical schools. Respondents under 30 years of age showed higher results (57.9%), followed by people between 30–39 years old (37.1%) and people aged between 40–49 years (28.8%). Participants > 50 years of age received minimum ethics education at medical school (19.4%).

No significant statistical differences regarding nationality and the type of ethics education were found except in medical schools, where Saudi Arabian physicians (41.3%) had a significant statistical difference *(P* = *0.007)* compared to non-Saudi Arabians (28%). However, in postgraduate programs, there was a significant statistical difference *(P* = *0.002)* between non-Saudi Arabians (11.9%) and Saudi Arabians (3.8%).

There was no significant statistical difference in relation to position or the type of ethics education, except that residents showed the highest agreement in relation to education in medical school *(P* = *0.00)*, followed by registrar/specialists and then consultants. The statement is correct regarding ethics education during sub-specialty programs *(P* = *0.00)* too.

No significant statistical difference was found between the type of board certificate and bioethics education, except for online training *(P* = *0.029)* and daily practice *(P* = *0.01).* The participants that received Western sub-specialist certificates had the highest agreement to learning from daily practice, while Saudi Arabian physicians had the least (Table 1).

Significant statistical difference was found in relation to the current workplace and bioethics education during residency programs *(P* = *0.015),* during sub-specialty programs *(P* = *0.04),* in postgraduate programs *(P* = *0.025)* and Online learning *(P* = *0.004)*. Online learning had a higher percentage of physicians who worked in private hospitals.

There was no significant statistical difference in relation to experience and the type of ethics education, except for education in medical schools *(P* = *0.00)* and during sub-specialty programs *(P* = *0.005)*. Physicians having experience of < 5 years showed the highest positive agreement followed by participants of 5–10 years, and then > 10 years. A high percentage of less experienced physicians had bioethics education in medical college, while those with more than 10 years' experience were found to have a significant statistical difference from those who had bioethics education through subspecialty training.

There was a significant statistical difference between the number of ethical challenges per month and bioethics education in medical school *(P* = *0.007),* in courses and workshops *(P* = *0.009)* and daily practice *(P* = *0.00).* Most of the respondents with ethics education from medical school and in courses and workshops had faced more than 10 challenges per month (47.60% and 28.60% respectively), whereas respondents with ethics education from daily practice had a maximum of 1–10 challenges per month (39%) (Table 1).

There was no significant statistical difference in relation to sub-specialty and the type of ethics education except for the general OB/GYN, which showed the least agreement compared to other types of subspecialties. The same finding is true regarding online training too.

#### Type of bioethics education and attitude towards ethical principles

No significant statistical difference was observed between the type of ethics education and ethical principles. Irrespective of the mode of ethics education, most of the respondents had a positive attitude towards various ethical principles. The highest positive attitude was towards respecting privacy of people and respecting confidentiality. Solidarity And Cooperation had the least positive attitude across all modes of ethics education (see Table 3).

**Table 3:** Respondents who received various form of ethics education and their attitude (Agree and Strongly Agree) towards the main four ethical principles

Type of Education			Autonomy		Non -maleficence		Beneficence		Justice		No stigmatization		Respect for cultural diversity and pluralism		Solidarity and cooperation	
			N	%	N	%	N	%	N	%	N	%	N	%	N	%
TOTAL			230	58.82	196	50.13	228	58.31	234	59.85	212	54.22	218	55.75	177	45.27
Formal Education	**Medical School**	**Yes**	80	58.39	72	52.55	82	59.85	78	56.93	80	58.39	80	58.39	67	48.91
		**No**	150	59.06	124	48.82	146	57.48	156	61.42	132	51.97	138	54.33	110	43.31
		**P**	0.899		0.481		0.650		0.388		0.223		0.440		0.289	
	**Residency**	**Yes**	27	58.70	22	47.83	28	60.87	26	56.52	23	50.00	22	47.83	18	39.13
		**No**	203	58.84	174	50.43	200	57.97	208	60.29	189	54.78	196	56.81	159	46.09
		**P**	0.985		0.740		0.707		0.625		0.541		0.251		0.372	
	**Sub-specialty**	**Yes**	11	64.71	10	58.82	10	58.82	11	64.71	11	64.71	10	58.82	9	52.94
		**No**	219	58.56	186	49.73	218	58.29	223	59.63	201	53.74	208	55.61	168	44.92
		**P**	0.612		0.463		0.965		0.675		0.373		0.794		0.517	
	**Postgraduate**	**Yes**	17	58.62	17	58.62	15	51.72	19	65.52	17	58.62	15	51.72	10	34.48
		**No**	213	58.84	179	49.45	213	58.84	215	59.39	195	53.87	203	56.08	167	46.13
		**P**	0.982		0.342		0.457		0.515		0.620		0.650		0.223	
Informal Education	**Conference**	**Yes**	44	59.46	39	52.70	48	64.86	45	60.81	39	52.70	38	51.35	31	41.89
		**No**	186	58.68	157	49.53	180	56.78	189	59.62	173	54.57	180	56.78	146	46.06
		**P**	0.902		0.623		0.202		0.851		0.771		0.397		0.516	
	**Online Training**	**Yes**	27	54.00	26	52.00	29	58.00	27	54.00	28	56.00	29	58.00	23	46.00
		**No**	203	59.53	170	49.85	199	58.36	207	60.70	184	53.96	189	55.43	154	45.16
		**P**	0.460		0.777		0.962		0.369		0.787		0.732		0.911	
	**Courses & Workshop**	**Yes**	31	59.62	26	50.00	33	63.46	31	59.62	28	53.85	27	51.92	21	40.38
		**No**	199	58.70	170	50.15	195	57.52	203	59.88	184	54.28	191	56.34	156	46.02
		**P**	0.901		0.984		0.417		0.971		0.954		0.551		0.446	
	**Daily Practice**	**Yes**	44	54.32	43	53.09	46	56.79	48	59.26	46	56.79	44	54.32	34	41.98
		**No**	186	60.00	153	49.35	182	58.71	186	60.00	166	53.55	174	56.13	143	46.13
		**P**	230		58.82		196		50.13		228		58.31		234	
	**Self-Learning**	**Yes**	68	54.84	59	47.58	71	57.26	67	54.03	69	55.65	69	55.65	56	45.16
		**No**	162	60.67	137	51.31	157	58.80	167	62.55	143	53.56	149	55.81	121	45.32
		**P**	0.276		0.492		0.773		0.111		0.598					

Type of Education			Social responsibility		Sharing of benefits		Protecting future generations		Protection of the environment		Respecting privacy of people		Respecting confidentiality	
			N	%	N	%	N	%	N	%	N	%	N	%
TOTAL			213	54.48	218	55.75	194	49.62	196	50.13	241	61.64	234	59.85
Formal Education	**Medical School**	**Yes**	77	56.20	80	58.39	69	50.36	73	53.28	84	61.31	80	58.39
		**No**	136	53.54	138	54.33	125	49.21	123	48.43	157	61.81	154	60.63
		**P**	0.614		0.440		0.828		0.359		0.923		0.667	
	**Residency**	**Yes**	21	45.65	23	50.00	21	45.65	19	41.3	28	60.87	28	60.87
		**No**	192	55.65	195	56.52	173	50.14	177	51.3	213	61.74	206	59.71
		**P**	0.202		0.404		0.567		0.202		0.909		0.88	
	**Sub-specialty**	**Yes**	10	58.82	10	58.82	11	64.71	9	52.94	12	70.59	12	70.59
		**No**	203	54.28	208	55.61	183	48.93	187	50	229	61.23	222	59.36
		**P**	0.712		0.794		0.203		0.812		0.433		0.35	
	**Postgraduate**	**Yes**	14	48.28	15	51.72	14	48.28	14	48.28	17	58.62	18	62.07
		**No**	199	54.97	203	56.08	180	49.72	182	50.28	224	61.88	216	59.67
		**P**	0.487		0.650		0.881		0.836		0.729		0.799	
Informal Education	**Conference**	**Yes**	38	51.35	40	54.05	33	44.59	33	44.59	50	67.57	47	63.51
		**No**	175	55.21	178	56.15	161	50.79	163	51.42	191	60.25	187	58.99
		**P**	0.549		0.744		0.337		0.29		0.241		0.473	
	**Online Training**	**Yes**	29	58.00	28	56.00	26	52	25	50	31	62	29	58
		**No**	184	53.96	190	55.72	168	49.27	171	50.15	210	61.58	205	60.12
		**P**	0.592		0.970		0.718		0.985		0.955		0.775	
	**Courses & Workshop**	**Yes**	26	50.00	29	55.77	22	42.31	22	42.31	29	55.77	30	57.69
		**No**	187	55.16	189	55.75	172	50.74	174	51.33	212	62.54	204	60.18
		**P**	0.487		0.998		0.257		0.226		0.353		0.734	
	**Daily Practice**	**Yes**	40	49.38	44	54.32	37	45.68	39	48.15	51	62.96	48	59.26
		**No**	173	55.81	174	56.13	157	50.65	157	50.65	190	61.29	186	60
		**P**	59.85		0.771		0.426		0.689		0.783		0.904	
	**Self-Learning**	**Yes**	66	53.23	64	51.61	59	47.58	61	49.19	73	58.87	71	57.26
		**No**	147	55.06	154	57.68	135	50.56	135	50.56	168	62.92	163	61.05
		**P**			0.262		0.583		0.801		0.444		0.477	

#### Type of bioethics education and attitude towards ethical challenges

The attitude of the OB/GYNs towards various ethical challenges in their daily practice were investigated. No statistical significance was observed between various forms of formal ethics education and ethical challenges, except there exists a significant statistical difference between post-graduate program and termination of pregnancy for non-medical *(P* = *0.05)* and between residency program and contraception issues *(P* = *0.021).* The respondents with postgraduate ethics education had a high positive response (agreed and strongly agreed, 31%) to the ethical challenge "Termination of pregnancy for a non-medical reason," and the respondents without residency program ethics education had a high positive response (agreed and strongly agreed, 46%) to the ethical challenge "contraception issues."

Pertaining to the informal mode of ethics education, significant statistical difference was observed between courses and workshops and paternity issues *(P* = *0.006)*; female consent *(P* = *0.004)*; breach of confidentiality *(P* = *0.007)*. There also exists a significant difference between breach of confidentiality and conference and workshop *(P* = *0.007)* and daily practice *(P* = *0.023)*. The respondents without courses and workshop mode of ethics education had agreed to the ethical challenges of paternity issues (33.92%), female consent (58%), and breach of confidentiality (33.6%). The respondents who did not have ethics education through conference (33%) and daily practice (33.8%) also agreed to the ethical challenge breach of confidentiality (Table 4).

**Table 4:** Respondents who received various form of ethics education and their attitude (agree and strongly agree) towards ethical challenges

Type of Education			Abortion for medical reason		Abortion for non-medical reason		Termination of pregnancy for medical reason		Termination of pregnancy for non-medical reason		Paternity issues		Contraception issues		Sex selection issues		Female consent	
			**N**	**%**	**N**	**%**	**N**	**%**	**N**	**%**	**N**	**%**	**N**	**%**	**N**	**%**	**N**	**%**
TOTAL			**245**	**62.66**	**68**	**17.39**	**245**	**62.66**	**66**	**16.88**	**123**	**31.46**	**172**	**43.99**	**117**	**29.92**	**216**	**55.24**
Formal Education	**Medical School**	**Yes**	81	59.12	22	16.06	82	59.85	23	16.79	47	34.31	61	44.53	37	27.01	72	52.55
		**No**	164	64.57	46	18.11	163	64.17	43	16.93	76	29.92	111	43.70	80	31.50	144	56.69
		**P Value**	0.289		0.608		0.4		0.972		0.374		0.875		0.353		0.433	
	**Residency Program**	**Yes**	26	56.52	6	13.04	26	56.52	7	15.22	10	21.74	13	28.26	16	34.78	24	52.17
		**No**	219	63.48	62	17.97	219	63.48	59	17.10	113	32.75	159	46.09	101	29.28	192	55.65
		**P Value**	0.363		0.396		0.363		0.746		0.123		0.021*		0.448		0.656	
	**Sub-specialty Program**	**Yes**	13	76.47	2	11.76	13	76.47	2	11.76	6	35.29	7	41.18	2	11.76	9	52.94
		**No**	232	62.03	66	17.65	232	62.03	64	17.11	117	31.28	165	44.12	115	30.75	207	55.35
		**P Value**	0.220		0.516		0.220		0.551		0.730		0.811		0.081		0.845	
	**Post Graduate Program**	**Yes**	16	55.17	7	24.14	17	58.62	9	31.03	7	24.14	10	34.48	10	34.48	16	55.17
		**No**	229	63.26	61	16.85	228	62.98	57	15.75	116	32.04	162	44.75	107	29.56	200	55.25
		**P Value**	0.390		0.334		0.642		0.050*		0.370		0.281		0.581		0.994	
Informal Education	**Conference**	**Yes**	47	63.51	9	12.16	46	62.16	9	12.16	18	24.32	28	37.84	17	22.97	38	51.35
		**No**	198	62.46	59	18.61	199	62.78	57	17.98	105	33.12	144	45.43	100	31.55	178	56.15
		**P Value**	0.866		0.177		0.922		0.218		0.137		0.235		0.141		0.455	
	**Online Training**	**Yes**	32	64.00	11	22.00	32	64.00	10	20.00	14	28.00	23	46.00	16	32.00	26	52.00
		**No**	213	62.46	57	16.72	213	62.46	56	16.42	109	31.96	149	43.70	101	29.62	190	55.72
		**P Value**	0.834		0.367		0.834		0.534		0.570		0.759		0.732		0.622	
	**Courses & Workshop**	**Yes**	32	61.54	7	13.46	31	59.62	6	11.54	8	15.38	17	32.69	10	19.23	19	36.54
		**No**	213	62.83	61	17.99	214	63.13	60	17.70	115	33.92	155	45.72	107	31.56	197	58.11
		**P Value**	0.858		0.412		0.627		0.255		0.006*		0.076		0.064		0.004*	
	**Daily Practice**	**Yes**	52	64.20	13	16.05	50	61.73	11	13.58	21	25.93	32	39.51	22	27.16	40	49.38
		**No**	193	62.26	55	17.74	195	62.90	55	17.74	102	32.90	140	45.16	95	30.65	176	56.77
		**P Value**	0.748		0.719		0.846		0.366		0.224		0.360		0.540		0.234	
	**Self-Learning**	**Yes**	80	64.52	23	18.55	76	61.29	22	17.74	39	31.45	55	44.35	35	28.23	66	53.23
		**No**	165	61.80	45	16.85	169	63.30	44	16.48	84	31.46	117	43.82	82	30.71	150	56.18
		**P Value**	0.605		0.682		0.703		0.757		0.999		0.921		0.617		0.585	

Type of Education			Adolescent marriages		Consanguinity marriages		Male doctor in OB/GYN field		Refusing to treat violated patients		Breach of confidentiality		Respecting Patient’s preferences		Inequality in patient’s care in response to their social grading	
			**N**	**%**	**N**	**%**	**N**	**%**	**N**	**%**	**N**	**%**	**N**	**%**	**N**	**%**
TOTAL			**125**	**31.97**	**150**	**31.46**	**187**	**47.83**	**91**	**23.27**	**122**	**31.20**	**220**	**56.27**	**151**	**38.62**
Formal Education	**Medical School**	**Yes**	47	34.31	51	37.23	58	42.34	34	24.82	46	33.58	76	55.47	53	38.69
		**No**	78	30.71	99	29.92	129	50.79	57	22.44	76	29.92	144	56.69	98	38.58
		**P Value**	0.468		0.734		0.110		0.597		0.458		0.817		0.984	
	**Residency Program**	**Yes**	15	32.61	17	36.96	22	47.83	11	23.91	14	30.43	20	43.48	20	43.48
		**No**	110	31.88	133	38.55	165	47.83	80	23.19	108	31.30	200	57.97	131	37.97
		**P Value**	0.921		0.834		1.00		0.913		0.905		0.064		0.473	
	**Sub-specialty Program**	**Yes**	5	29.41	6	35.29	10	58.82	3	17.65	6	35.29	13	76.47	6	35.29
		**No**	120	32.09	144	38.50	177	47.33	88	23.53	116	31.02	207	55.35	145	38.77
		**P Value**	0.816		0.789		0.354		0.566		0.712		0.082		0.773	
	**Post Graduate Program**	**Yes**	10	34.48	12	41.38	14	48.28	6	20.69	9	31.03	16	55.17	13	44.83
		**No**	115	31.77	138	38.12	173	47.79	85	23.48	113	31.22	204	56.35	138	38.12
		**P Value**	0.764		0.729		0.960		0.730		0.984		0.902		0.478	
Informal Education	**Conference**	**Yes**	17	22.97	26	35.14	35	47.30	14	18.92	16	21.62	36	48.65	33	44.59
		**No**	108	34.07	124	39.12	152	47.95	77	24.29	106	33.44	184	58.04	118	37.22
		**P Value**	0.061		0.525		0.919		0.318		0.044*		0.144		0.243	
	**Online Training**	**Yes**	13	26.00	21	42.00	27	54.00	11	22.00	18	36.00	29	58.00	20	40.00
		**No**	112	32.84	129	37.83	160	46.92	80	23.46	104	30.50	191	56.01	131	38.42
		**P Value**	0.327		0.573		0.350		0.819		0.437		0.791		0.830	
	**Courses & Workshop**	**Yes**	12	23.08	17	32.69	23	44.23	8	15.38	8	15.38	24	46.15	18	34.62
		**No**	113	33.33	133	39.23	164	48.38	83	24.48	114	33.63	196	57.82	133	39.23
		**P Value**	0.133		0.363		0.577		0.137		0.007*		0.116		0.522	
	**Daily Practice**	**Yes**	20	24.69	34	41.98	38	46.91	16	19.75	17	20.99	41	50.62	29	35.80
		**No**	105	33.87	116	37.42	149	48.06	75	24.19	105	33.87	179	57.74	122	39.35
		**P Value**	0.110		0.454		0.853		0.395		0.023*		0.251		0.558	
	**Self-Learning**	**Yes**	43	34.68	48	38.71	57	45.97	35	28.23	39	31.45	70	56.45	51	41.13
		**No**	82	30.71	102	38.20	130	48.69	56	20.97	83	31.09	150	56.18	100	37.45
		**P Value**	0.435		0.924		0.616		0.118		0.942		0.960		0.488	

^*^Significant at α level less than 0.05

## Discussion

There are approximately similar percentages of participants who received formal ethical education, never had formal ethical education and who received mixed type of ethical education. The percentages of these ranged between 18.0% -22%. 18.9% percentage of respondents received formal ethics education out of which 35% received it in medical school, whereas 31.7% practiced self-learning as an informal form of ethics education. There was a significant lack of formal education in residency, postgraduate and subspecialty programs with 11.8%, 7.4% and 4.3%, respectively. Although a study by *(Byrne et.al. 2015*) [38] emphasized a greater need for ethics education in obstetrics-gynecology programs, they still reported 50.4% of cohorts had received formal ethics education in their residency programs.

Albeit female respondents were more than male respondents, a greater percentage of male respondents had received ethics education (both formal (16.9%) and informal (23.4%)) than the female respondents (13.6%, 17.7% respectively).

The demographics revealed that about 57% of young respondents of < 30 years of age in OB/GYN specialty have received ethics education in medical school. The percentage decreased to 19% as the age of participants increased to > 50 years. This could be subsequent to the recent introduction of ethical education in various medical colleges in Saudi Arabia and around the world [39].

The Saudi Arabian physicians (41.3%) had a significant statistical difference *(P* = *0.007)* than non-Saudi Arabian (28%) when comparing ethics education in medical schools. This supports the effect of the implementation of ethics education during undergraduate years. However, this finding is diminished in post-graduation due to a lack of ethics education in the OB/GYN residency programs. The study depicted a significant statistical difference *(P* = *0.002)* in non-Saudi Arabian (11.9%) vs Saudi Arabian (3.8%) post-graduation ethics education. Other researchers reported insufficient ethics teaching in different OB/GYN Boards, too [40]. Nonetheless, they had a higher percentage of formal ethics education in their curricula than reported in this study. A survey among undergraduate medical students by *(Mufti *et al*., 2022)* [41] states that though 76.7% of the participants considered ethical knowledge very important, about 64.5% of the participants had a poor level of knowledge of ethics.

An increased number of married respondents had ethics training through their daily medical practice, whereas others practiced self-learning as means of ethics training. Saudi Arabian Board respondents had a significant statistical difference *(P* = *0.01)* for learning from daily medical practice than others. The participants who received Western sub-specialty certificates had the highest agreement on learning from online training, while the Saudi Arabian physicians had the least agreement.

Despite the higher number of Muslim respondents, a higher percentage of non-Muslim respondents had received various modes of formal ethics education except for postgraduate programs, where none of the non-Muslim respondents agreed o it. But on average, about 14.1% and 19.26% of Muslim respondents and 26.9% and 26.18% of non-Muslim respondents had received formal and informal modes of ethics education, respectively.

The workplace had a significance with ethics education. The respondents from the governmental non-teaching hospital had received a higher percentage of ethics education from residency programs (26.9%) than the respondents from the private hospitals (10.9%). But on average, a higher percentage of the respondents from private hospitals had received both the formal and informal mode of ethics education. This was in contrast to the study reported by *(Alharabi *et al*., 2018*) [42], where it was observed that about 92.3% of the government hospital physicians had studied bioethics and 84.4% had an agreement to ethics application.

A high percentage of less experienced respondents had bioethics education in medical college, whereas those with experience of > 10 years showed a significant statistical difference in terms of bioethics education through subspecialty training.

Most of the participants (79%) rarely face ethical challenges (less than one challenge/month), which clearly refers to the critical need of ethics education. Especially a higher percentage of participants who had received ethics education from medical schools encounter more than 10 ethical challenges per month (47.6%). The same was the case with courses and workshops and daily practice. On average, about 23.8% of the respondents with formal education encounter more than 10, and about 28.2% of the respondents with informal education encounter about 1–10 ethical challenges per month. This can be an outcome of an unstructured ethics curriculum that fails to enhance the competence to deal with ethical challenges in daily practice. The objective of ethics education is to develop the physicians' skills to analyze and resolve ethical dilemmas. The philosophical lectures taught within classrooms alone cannot make medical students ethically competent [31]. The survey reported by (*Aldughaither *et al*., 2012)* [43] states that more than 85% of the participants considered that the method of instruction in ethics education is not effective. Though lecturing is the most common form of ethics education in medical school [44], students prefer that the method has to be focused on case-based teaching. About 66.8% and 59.6% of the medical students preferred that ethics education has to focus on abortion and reproduction challenges, respectively [43].

In this survey, there is no significant statistical difference in relation to position and type of ethical education except for ethics education in medical school and sub-specialty programs. As mentioned earlier, the resident respondents showed the highest agreement regarding education in medical school, followed by registrar/specialist and then consultants. The same is correct for ethics education in sub-specialty programs regarding consultants in sub-specialty. However, male respondents agreed to have received ethical education in sub-specialty programs more than female respondents. The same may be implied for self-learning and online training.

Informal education enveloped self-learning, attending conferences, courses, workshops, and daily practice implemented by 13–20% of the participants. In reality, it mirrors the mindfulness of ethical knowledge in daily medical practice and the urgent need for reinforcement of ethics education not only in Saudi Arabia but globally [39, 45, 46]. Online education as a method of ethical education is an established method of training for the medical fraternity; therefore, it is imperative to be well aware of emerging ethics education resources [47].

Respondents from different modes of ethics education had almost similar positive attitudes towards various ethical principles. This shows that ethics education has incorporated the values, importance, and knowledge of ethical principles.

We next investigated the statistical significance between the mode of ethics education and various ethical challenges and principles. Most of the respondents, irrespective of the mode of ethics education, considered abortion and termination of pregnancy for medical reasons (62.6%) as ethically challenging. Abortion (17.4%) and termination of pregnancy (16.9%) for non-medical reasons were considered as the least ethically challenging issues, except that a higher percentage of respondents with postgraduate ethics education considered termination of pregnancy for non-medical reason as ethically challenging (31%, *P* = *0.05*). This was in contrast with the survey reports by (*Alhumaid *et al*., 2023*) [48] where the survey was conducted among medical students. This survey states that more than 50% of the medical students were satisfied with the ethics education regarding the ethical issues surrounding abortion. Most of the participants supported abortion in case of medical reasons (cases of endangered mother's life, fetal life compromise) and were against non-medical reasons (cases of financial incapacity of the parents and cases of unplanned pregnancy). This can be because of the differences in theoretical ethics education and real-time ethical challenges in medical practice. This is in line with the finding reported by *(Alardan *et al*., 2021)* [49], which states that there exists a gap between knowledge, attitude, and practice of medical ethics and emphasizes strengthening medical ethics education in Saudi Arabia. A well-structured curriculum, trained mentors, and a standardized mode of teaching are needed to bridge the gap [30].

There was a significant statistical difference between the respondents who received ethics education from courses and workshops and various ethical challenges. The higher percentage of respondents who have not attended courses and workshops considered paternity issues (33.9%), female consent (58%) and breach of confidentiality (33.6%) as ethically challenging when compared to the respondent who had attended courses and workshops. Breach of confidentiality also had significant statistical differences across various modes of informal ethics education such as conferences, courses and workshops and daily practice.

## Conclusion

The low rate of receiving bioethics education among obstetricians and gynecologists raises serious concerns about the quality of care and relationships with patients. It highlights a glaring need for bioethics education in this field in Saudi Arabia.

The need for teaching extends beyond formal education at the undergraduate and postgraduate levels. It is essential to recognize that learning is a lifelong process, and OBGYN physicians require continuous informal education and development throughout their lives.

It is crucial to prioritize teaching diverse ethical principles and provide comprehensive training on ethical challenges and their practical applications in clinical practice. This approach ensures that OBGYN physicians are equipped with the necessary knowledge and skills to navigate complex ethical dilemmas they may encounter in their day-to-day work.

Implementing bioethics educational programs requires diverse teaching methods to achieve their goals effectively. These methods include direct lectures, workshops, clinical case discussions, and bedside ethics teaching. By combining these various teaching methods, educators can create dynamic and comprehensive learning experiences for aspiring OBGYN physicians in the field of bioethics, especially when following a longitudinal theme.

When developing bioethics educational programs for OBGYN physicians in Saudi Arabia, it is crucial to consider universal ethics and local perspectives. While universal ethics provide a foundation for ethical principles applicable across different cultures and societies, it is equally important to consider the unique cultural and religious context of Saudi Arabia.

Further research is essential to build the best content and develop effective teaching methods. This research should focus on understanding the needs and preferences of Saudi OBGYN physicians, as well as identifying the most appropriate teaching techniques.

## Limitations

This research has many limitations. Some critical issues were not included in our study, such as training for medical error reporting & the proper methods of teaching bioethics to OBGYN doctors in Saudi.

Simple surveys were used in this study. However, different types of research will be needed to have a clear image to investigate the issues related to bioethics education. These methods may include case analysis and qualitative research.

### Supplementary Information

**Additional file 1.**

## Data Availability

The datasets used and/or analyzed during the current study are available from the corresponding author upon reasonable request.
