# Supplementary material for: Ethics education among obstetrics and gynecologists in Saudi Arabia: a cross-sectional study
Source: BMC Med Educ. 2023 Nov 16;23:872. doi: 10.1186/s12909-023-04824-x (PMC10655446; doi:10.1186/s12909-023-04824-x)
Supplement: Supplementary file 1 — Additional file 1. [file 12909_2023_4824_MOESM1_ESM.docx]

**Questioners**

**First Part:**

**Demographics and training in ethics**

**A) Demographic data:**

Age Sex 1) F 2) M

Marital status: 1) Married 2) Divorced 3) Widow 4) Conjugal 5) Non-Married

Nationality: 1) Saudi 2) Non-Saudi Religion: 1) Muslim 2) Non-Muslim

Medical school from which you get your MBBS or MD:

Year of experience (total) Years of experience in Saudi Arabia

Your current workplace: 1) Tertiary and teaching hospital 2) governmental and teaching hospital

3) Private hospital. 4) Governmental non-teaching hospital

Your current position: 1) Resident, 2) Registrar (or specialist), 3) Consultant

If you are a registrar (or specialist) or consultant:

- What is your Board Certificate? Check all that may be applied

1) Arab Board, 2) Saudi Board, 3) Egyptian (Master, PhD)

4) Jordanian Board 5) Canadian Board 6) MRCOG

7) ABOG 8) Other

**B) Bioethics education and training:**

**Did you receive teaching in bio-medical ethics?**

1) Formal education 2) Non formal education 3) Both 4) None of them

**If you have formal education, where did you receive it?**

1) In medical school. 2) During residency programs.

3) During Subspecialty programs. 4) In postgraduate programs: (Diploma, Master, PHD)

**If you have nonformal education, how did you receive it?**

1) In conferences. 2) Online training. 3) In courses and workshops.

4) Daily practice (Grand round, case presentation, and committee). 5) Self learning.

**Second Part:**

**Bioethics principles**

This section asks for your opinion regarding some principles of medical ethics.

The following ethics principles are important in OB/GYN practice. Please respond to the following statements, by providing one of choices, from 1 to 5, as following: (1-strongly disagree, 2-disagree, 3-not sure, 4-agree and 5-strongly agree).

| The following ethics principles are important in OB/GYN practice | Strongly disagree | Disagree | Not sure | Agree | Strongly agree |
| --- | --- | --- | --- | --- | --- |
| Autonomy |  |  |  |  |  |
| Non maleficence |  |  |  |  |  |
| Beneficence |  |  |  |  |  |
| Justice |  |  |  |  |  |
| No discrimination |  |  |  |  |  |
| No stigmatization |  |  |  |  |  |
| Respect for cultural diversity and pluralism |  |  |  |  |  |
| Solidarity and cooperation |  |  |  |  |  |
| Social responsibility |  |  |  |  |  |
| Sharing of benefits |  |  |  |  |  |
| Protecting future generations |  |  |  |  |  |
| Protection of the environment |  |  |  |  |  |
| Respecting privacy of people |  |  |  |  |  |
| Respecting confidentiality |  |  |  |  |  |

**Third Part:**

**Ethically challenging issues in OB/GYN practice**

This section asks for your opinion regarding some ethical issues in OB/GYN practice.

The following issues are ethically challenging in OB/GYN practice. Please respond to the following statements, by providing one of choices, from 1 to 5, as following: (1-strongly disagree, 2-disagree, 3-not sure, 4-agree and 5-strongly agree).

| The following issues are ethically challenging in OB/GYN practice in General | Strongly disagree | Disagree | Not sure | Agree | Strongly agree |
| --- | --- | --- | --- | --- | --- |
| Abortion for medical reason. |  |  |  |  |  |
| Abortion for non-medical reason |  |  |  |  |  |
| Termination of pregnancy for medical reason |  |  |  |  |  |
| Termination of pregnancy for non-medical reason |  |  |  |  |  |
| Paternity issues |  |  |  |  |  |
| Contraception issues |  |  |  |  |  |
| Sex selection issues |  |  |  |  |  |
| Female consent |  |  |  |  |  |
| Adolescent mariages |  |  |  |  |  |
| Consanguinity marriages |  |  |  |  |  |
| Male doctor in OB/GYN field |  |  |  |  |  |
| Refusing to treat violated patients |  |  |  |  |  |
| Breech confidentiality |  |  |  |  |  |
| Respecting Patient’s preferences |  |  |  |  |  |
